# Satisfaction of inpatients with acute coronary syndrome in Bulgaria

**DOI:** 10.1186/1477-7525-6-50

**Published:** 2008-07-14

**Authors:** Milka Ganova-Iolovska, Krassimir Kalinov, Max Geraedts

**Affiliations:** 1 National Center of Public Health Protection, 15, Ivan Ev. Geshov Blvd, 1341, Sofia, Bulgaria; 2 New Bulgarian University, Department of Computer Science, 21, Montevideo street, 1618, Sofia, Bulgaria; 3 Public Health Programme, University Hospital of the Heinrich-Heine-University, Moorenstraße 5, 40225, Düsseldorf, Germany

## Abstract

**Background:**

Patient satisfaction constitutes an important indicator for the quality of care. During the last years, Bulgaria changed its socialist health care system to a market-driven system. Despite the fact that the improvement of health care quality and patient satisfaction were put on top of the list of goals for the health care reforms, no studies of patient satisfaction with inpatient care have been conducted so far.

Since cardiovascular diseases are amongst the major causes of death in Bulgaria, and strenuous efforts have been made to improve the quality of medical care of patients with acute coronary syndrome (ACS) during the last years, patient satisfaction in this group can be seen as an important example of the Bulgarian reforms. This study therefore investigates patient satisfaction of inpatients with ACS.

**Methods:**

We performed structured face-to-face interviews with all patients with ACS, residing in a representative Bulgarian region who were discharged from hospitals in this region between September 1st and December 31st, 2004. We surveyed their socio-demographic status, overall satisfaction, change in complaints, self-perceived health status, functional possibilities in activities of daily living, satisfaction with life and self-reported condition at admission. We used descriptive methods as well as t-tests, chi-square tests, and logit models for data analysis.

**Results:**

Face-to-face interviews were carried out in 394 cases, of which 53.6% were men and 46.4% were women. 24% of the patients were satisfied with inhospital treatment, 62% were satisfied to some extent, and 14% were unsatisfied. The overall satisfaction of patients with ACS was significantly associated (p < 0.05) with the type of hospital, the number of family members living together and the severity of the disease at admission. Patients treated in urban and middle-size rural hospitals, patients living together with three or more family members, and patients with more severe conditions at admission reported higher satisfaction scores.

**Conclusion:**

ACS patient satisfaction with inhospital treatment in Bulgaria shows much room for improvement. Information obtained from satisfaction studies could be used at decision-making and hospital-management levels for improving new strategies and structural changes in the Bulgarian health care system.

## Background

Evaluation of the quality of health care is a complex and challenging process. Currently, there is an emphasis on the use of outcome indicators as a measurement of the quality of health care. Patient satisfaction is a category that has received attention as a useful indicator of the quality of care in consumer-driven health care systems. Measurement of patient judgments about quality of inpatient care and health outcomes is advancing rapidly worldwide, mainly for to two reasons: First of all, patients are in an excellent position to evaluate certain aspects of the process of care. Secondly, learning about what consumers want from their health care system and what quality care means to them offers decision-makers a better understanding of their expectations.

Patient satisfaction has been defined as the degree of congruency between a patient's expectations of ideal care and his or her perception of the real care he or she receives [1]. It is a perceptional process that is sometimes associated with several socio-demographic variables, such as age, sex, the level of education, employment, income or marital status [2-7]. Therefore, patient satisfaction is a subjective perception from the patient's point of view that caregivers can regard as reality, even though this perception may disregard the appropriateness of therapies and outcomes of the patient's health status [7-9].

During the last eight years, Bulgaria changed its old socialist health care system to a new, decentralized, market-driven and patient-centered system. Comparable to many countries in Europe, health care quality improvement and patient satisfaction are amongst the cornerstones of the reform goals.

Despite the fact that patient satisfaction forms one of the main goals of the new Bulgarian health care system, no studies evaluating inpatient satisfaction as an important indicator for outcome quality have been conducted until now. Therefore, the aim of our study was to evaluate patient satisfaction with inpatient care in patients with ischaemic heart disease – the main cause of disability and death in Bulgaria – in a region typical for Bulgaria and to assess the influence of certain socio-demographic factors, treatment characteristics and individual perceptions on patient satisfaction.

## Methods

### Study region

The survey was carried out in the Stara Zagora region which is typical for Bulgaria with its demographic (age, sex and urban/rural distribution) and health care characteristics. The region includes almost 5% of the total Bulgarian population of about 7.8 million people [10,11]. Inpatient care of patients with ischaemic heart diseases is provided in all hospitals of the region – one university clinic, one regional and four community hospitals. All hospitals in the region provide the same cardiology diagnostic and treatment approaches that do not differ from average treatment provided in Bulgaria.

### Target population

In Bulgaria, as in many parts of the world, cardiovascular diseases (CVD) present the main cause of death and disability. CVD accounted for 61.5% of all deaths in Bulgaria in 1990 and for 67.5% of all deaths in 2004 [12,13]. In 2004, 16.7% of all deaths were due to ischaemic heart diseases (IHD) and 6.4% of them to acute myocardial infarction (AMI). Because of the importance of the IHD, we chose all patients with acute coronary syndrome (ACS) as our study population.

All patients residing in the Stara Zagora region that were admitted and treated at any of the six hospitals of the region with ACS during the period from September 1st, 2004 to December 31st, 2004 were registered.

Since there are no ethics committees in Bulgaria, the study was approved by the Ministry of Health for its concordance with the ethical standards accepted in Bulgaria (Declaration of Helsinki and the Convention for security on the rights of the human's dignity from 1996). Furthermore the executive hospital bodies were acquainted with the study protocol and their permission for conducting the study was obtained as well.

Every patient with ACS was visited by an interview-team member and received verbal and written information about the design and goals of the study during their inpatient stay. A day before discharge, a second visit was undertaken. If the patient agreed to take part in the study, a written consent for voluntary participation was obtained.

### Instrument

We adopted the *FK-P *questionnaire, developed and verified by the Department of Medical Sociology of the University Medical Centre Hamburg-Eppendorf [14,15] with additional questions from the questionnaire *2000 KPF *developed and implemented by the Department of Medical Sociology of the Institute for Occupational and Social Medicine of the University of Cologne [16-18]. We included four aspects in the questionnaire *FK-P *(accommodation, attitude towards patient opinion, physician care, and coordination) with 2 to 3 questions per aspect from the questionnaire *2000 KPF *in order to capture some additional aspects of inpatient care in Bulgaria.

Via back-translation techniques, the instrument was translated into Bulgarian and then back again into German. Two different translators independently completed the initial and back-translation. The back-translated version was compared with the original German one by a third translator and checked for conceptual discrepancies. Additionally, a pilot test aiming at detection of potential problems was conducted amongst ACS-inpatients in the Stara Zagora region [19-24].

We adopted the five-point Likert scale from the *FK-P *questionnaire. The scale is numbered from 1 (do not agree) to 5 (strongly agree). We added the answer option "I can not evaluate it" to all questions excluding personal data, self-perception, disease severity and overall satisfaction with the episode of inpatient care. The reason for changing the scale was primarily the assumption that Bulgarian patients were not used to evaluate the hospital stay. This is because, until 2001, Bulgarian patients seldomly were requested to express their satisfaction with medical care and due to cultural and historical reasons, Bulgarians were habitually grateful to healthcare providers and were not used to express criticism towards them. If patients replied with the option "I can not evaluate it", these answers were treated as missing values.

For the item "monthly income", we included the option "I don't want to give an answer".

The final Bulgarian questionnaire included socioeconomic status (SES) and different aspects of inpatient care – 1) admission, 2) accommodation, 3) attitude towards patient opinion and participation in decision making, 4) nursing care, 5) physician care, 6) care provided by other medical staff, 7) internal coordination, 8) information about the disease, the treatment approaches, and achieved medical goals, 9) education and discharge information, and 10) care after discharge.

### Interview setting

All patients were interviewed by trained interviewers in a structured face-to-face interview conducted between two to four weeks after discharge at the patient's place of living. The interviewers were trained at the National Centre of Public Opinion. We selected as interviewers local residents from Stara Zagora region who were not employed in medical institutions. For the aim of the study, the interviewers received additional training.

### Key measures

In the analysis, the principal measure was overall satisfaction with inpatient care. Predictors included SES (age, gender, education, employment status, personal monthly income, marital status, household size), hospital type, length of stay (LOS) as well as incidents of acute myocardial infarction (AMI) and/or angina pectoris (AP) in the past. In addition, we analyzed the patient's self-evaluation of his or her health, the change in compliance, the ability to perform activities of daily living, the satisfaction with life and the self-reported heaviness of condition at admission as variables related to the achievement of treatment goals.

### Analytical model

Due to the small number of cases, we combined the university and regional hospital data as one group, while the group of middle-sized rural and the group of small community rural hospitals formed two more groups.

We used a multiple logistic regression model as an analytical tool. All predictors that were significantly associated to the dependent variable at a level of significance of 0.05 (chi-square tests) were consecutively put into the model. The influence of the variables in the model was estimated by odds ratios and 95% confidence intervals.

We also computed Pearson's correlation coefficients to determine the level and the direction of linear relationships between overall satisfaction and the aspects of inpatient care included in the questionnaire.

## Results

412 patients residing in the Stara Zagora region were discharged during the period between September 1^st^, 2004 and December 31^st^, 2004 with the main diagnosis of ACS. 16 patients (4%) rejected the participation in the study (96.1% cooperation rate) naming various reasons such as lack of time or simply unwillingness to participate in the study. Two patients died at home during the first days after discharge (95.6% participation rate).

Face-to-face interviews were carried out in 394 cases, of which 53.6% were men and 46.4% were women at an average of 19 days (median 18 days, SD 5.4) after discharge. 54% of men and 46% of women were younger than 65 years of age. The demographic characteristics and parameters of the socio-economic status are summarized in Table 1.

**Table 1:** Basic characteristics of the study population

**Basic characteristics**		**N**	**Percentage**
**Gender**	male	211	53.6
	female	183	46.4
**Age**	≤ 64 years	213	54.1
	≥ 65 years	181	45.9
**Education**	primary school or less	174	44.2
	secondary school	169	42.9
	college or high school	51	12.9
**Employment status**	unemployed	305	77.4
	employed	89	22.6
**Personal monthly income**	≤ 51 €	105	27.3
	51.1 – 102 €	187	48.7
	102.1 – 153 €	48	12.5
	≥ 153.1 €	26	6.8
	unemployed without unemployment benefits	1	0.3
	answer denied	17	4.4
**Marital status**	married/partner	127	32.2
	single	267	67.8
**Family members**	1 member	87	22.1
	2 members	202	51.3
	3 members	57	14.5
	4 or more members	48	12.2
**Type of hospital**	regional center	180	45.7
	medium sized town	153	38.8
	small town	61	15.5
**Length of stay**	≤ 8 days	286	72.6
	≥ 9 days	108	27.4
**AMI and/or AP in the past**	no	227	57.6
	AMI and/or AP	167	42.4

**Total**		394	100.0

Table 2 depicts the distribution of the predictor variables as frequencies and percentages together with the distribution of overall satisfaction.

**Table 2:** Self-reported conditions and overall satisfaction

**Success of treatment from patient's perspective**		**N**	**Percentage**
**Overall satisfaction with inpatient care**	unsatisfied	54	13.7%
	somewhat satisfied	241	62.4%
	satisfied	94	23.9%

**Self-perceived health status**	poor	20	5.1%
	not very good	204	51.8%
	good	160	40.6%
	very good	10	2.5%

**Change in complaints**	worsened	16	4.1%
	not changed	58	14.7%
	improved	320	81.2%

**Ability to perform activities of daily living**	poor	49	12.4%
	good	85	21.6%
	very good	260	66.0%

**Satisfaction with life**	not at all satisfied	205	52.0%
	not very satisfied	171	43.4%
	very satisfied	18	4.6%

**Self-reported condition at admission**	not very severe	19	4.8%
	averagely severe	113	28.7%
	pretty severe	164	41.6%
	very severe	98	24.9%

**Total**		394	100.0%

24% of the patients were satisfied with the treatment in the hospital setting, 14% were unsatisfied and 62% of the patients were satisfied to some extent.

Nearly 82% of the patients reported an improvement of their complaints and 88% indicated that their ability to perform activities of daily living after treatment were good or very good. 43% of the patients reported a good or very good health status after discharge. Only 5% of the study population reported to be very satisfied with their life.

Chi-square tests showed several factors (severity of the disease, hospital type and number of family members) to be significantly associated to the dependent variable "overall satisfaction with inpatient care" (Table 3).

**Table 3:** Chi-square tests of factors influencing overall satisfaction with inpatient care in Bulgarian ACS patients

**Variables**		**N**	**%**	**Sig**
**Overall satisfaction with** **inpatient care**	unsatisfied	54	13.7%	
	somewhat satisfied	246	62.4%	
	satisfied	94	23.9%	

**Gender**	male	211	53.6%	0.083
	female	183	46.4%	

**Self-reported condition ** **at admission**	not very severe	19	4.8%	0.012
	somewhat severe	113	28.7%	
	pretty severe	164	41.6%	
	very severe	98	24.9%	

**Type of hospital**	regional center	180	45.7%	0.000
	medium sized town	153	38.8%	
	small town	61	15.5%	

**Household size**	1 member	87	22.1%	0.034
	2 members	202	51.3%	
	3 members	57	14.5%	
	4 or more members	48	12.2%	

**Valid**		394	100.0%	

**Missing**		0		

**Total**		394	100.0%	

Using the category "satisfied" as a reference of the dependent variable, the logistic regression model was used once more. In the multivariable analysis, satisfaction with inpatient care was significantly related to the type of hospital (urban and medium rural), the number of family members living together and the severity of the disease at admission from the patient's point of view (Table 4).

**Table 4:** Odds ratios (OR) and 95% confidence intervals (CI) of the factors influencing satisfaction with inpatient care in Bulgarian ACS patients

**Satisfaction**	**Variables**	**Regression** **coefficient**	**Standard** **error**	**Sig.**	**OR**	**95% C I**	
						
						**lower**	**upper**
**Unsatisfied**							

	Self-reported condition at admission						
	not very severe	-1.592	0.946	0.092	0.203	0.032	1.299
	average severe	-0.886	0.545	0.104	0.412	0.142	1.200
	pretty severe	0.174	0.475	0.715	1.190	0.469	3.016
	very severe	0					
	Hospital type						
	urban	-3.546	0.793	0.000	0.029	0.006	0.137
	medium rural	-4.439	0.842	0.000	0.012	0.002	0.061
	small rural	0					
	Family members						
	1 member	0.846	0.686	0.218	2.329	0.607	8.934
	2 members	1.222	0.632	0.053	3.393	0.982	11.722
	3 members	1.508	0.730	0.039	4.516	1.081	18.870
	4 or more	0					

**Somewhat satisfied**							

	Self-reported condition at admission						
	not very severe	-1.885	0.592	0.001	0.152	0.048	0.484
	averagely severe	-0.255	0.349	0.465	0.775	0.391	1.536
	Pretty severe	0.028	0.331	0.933	1.028	0.538	1.965
	very severe	0					
	Hospital type						
	urban	-2.08	0.756	0.007	0.132	0.030	0.583
	medium rural	-2.055	0.758	0.007	0.127	0.029	0.563
	small rural	0					
	Family members						
	1 member	0.768	0.413	0.063	2.156	0.960	4.841
	2 members	1.214	0.372	0.001	3.367	1.625	6.978
	3 members	0.827	0.468	0.077	2.287	0.915	5.720
	4 or more	0					

The Pearson's correlation test showed a moderate positive correlation between overall satisfaction and satisfaction with different aspects of inpatient care between 0.594 and 0.163 at a level of significance of 0.01 (Table 5).

**Table 5:** Correlation between overall satisfaction and different aspects of inpatient care

	**Aspects of inpatient care**									
	
	**1**	**2**	**3**	**4**	**5**	**6**	**7**	**8**	**9**	**10**
*PCC	.163(**)	.474(**)	.422(**)	.454(**)	.397(**)	.217(**)	.477(**)	.498(**)	.594(**)	.258(**)
**Sig.(2-tailed)**	.001	.000	.000	.000	.000	.000	.000	.000	.000	.000

1. *PCC – Pearson's' correlation coefficient

2. (**) – significant at 0.01

3. aspects – 1) admission, 2) accommodation, 3) attitude towards patient opinion and participation in decision making, 4) nursing care, 5) physician care, 6) care provided from other medical staff, 7) internal coordination, 8) information about the disease, the treatment approaches and achieved medical outcomes 9) education and information for discharge, 10) care after discharge

Overall satisfaction correlated positively in particular with education and with information about potential complications and health-related behaviour after discharge (0.594). Overall satisfaction also correlated with information about the disease, treatment approaches, achieved medical goals (0.498) and internal coordination (0.477).

## Discussion

ACS patient satisfaction with inpatient care in Bulgaria shows much room for improvement and is associated with the type of hospital, the number of family members living together and the severity of the disease at admission. Our findings suggest that Bulgarian male inpatients and individuals living in big families tend to be more satisfied with hospital care. Patients reporting their condition at admission as severe are more satisfied with inpatient care. From the patient's point of view, particularly urban and middle rural hospitals fulfill their expectations of quality health care.

We measured patient satisfaction by using, for the first time in Bulgaria, internationally accepted methods and were able to demonstrate that a measurement of inpatient satisfaction is indeed possible in Bulgaria. The information obtained from satisfaction studies could be used at the hospital-management and health care system levels to improve strategies, structures and processes of care in Bulgaria.

Concerning the generalization of our results from the region Stara Zagora to Bulgaria, it has to be acknowledged that socio-demographic patterns of the study region's population are comparable to Bulgaria. Diagnostic and treatment approaches for ACS patients are also similar and the level of care provided by the study hospitals corresponds to the Bulgarian average.

The methods we used followed generally accepted rules in that we used an instrument based on two validated patient satisfaction questionnaires from Germany that were correctly translated. The interviews took place outside the hospitals within an adequate period after discharge [9] and the interviewers were not members of the hospital staff.

Nevertheless, our findings are not in conformity with several studies on the topic. Studies by Powers et al. and Chang et al. for instance show demographic characteristics such as age and sex and the socio-economic status (education, employment, income, marital status, number of family members living together) to be generally related to patient satisfaction [3,4]. Studies carried out in Eastern European countries reported similar results [25].

In the region of Stara Zagora, the socio-demographic variables age, education level, employment status, personal monthly income and marital status did not significantly influence patient satisfaction. We only found a tendency suggesting men being more satisfied with inpatient care than women. Comparable results have been reported by several studies [9,15,26,27]. In 2002, Crow et al. analysed the results of 39 studies and reported that a firm conclusion about the relationships between reported satisfaction and gender cannot be drawn [28].

As opposed to findings by Hall, we found that Bulgarians living in bigger families were more satisfied with inpatient care than those in smaller families [6]. Our results suggest that those patients could be less demanding than subjects living in smaller families. The fact that Bulgarians with bigger families usually have more responsibilities for their relatives and strive for a quicker return to their work place and/or home may additionally influence their responses.

Jenkinson et al. have reported that about 90% of inpatients were satisfied with the episode of care [29]. In the region of Stara Zagora, 24% of ACS patients were very satisfied and 62% were satisfied to some extent with inpatient treatment. Comparable percentages of patients reported that their complaints and their ability to perform activities of daily living improved. At the same time, nearly 57% of the patients reported poor or not very good health and 52% reported that they were not at all satisfied with their life. Since some authors suggest that sick and depressed patients tend to rate patient satisfaction worse, the Bulgarian patient satisfaction may be influenced to a great extend by this factor [7,9,30].

Moreover, we tested some variables concerning medical care during the actual inpatient episode of care such as the length of stay, the therapeutic success and the patient's self-reported severity of condition as well as additional predisposing factors such as the history of IHD and the satisfaction with life. Our findings show that in the region of Stara Zagora, only the self-reported severity of the condition at admission was significantly positive associated with patient satisfaction. Comparable results have been published by Thi et al. and could be explained with the effectiveness of medication in the inpatient setting [9]. However, our study did not replicate the findings by Thi et al. and Perneger, who showed that patient satisfaction was dependent on the length of stay and their medical history [9,30].

Young et al. reported that institutional characteristics such as size, teaching status and location of hospitals were associated with patient satisfaction [5]. For medium rural hospitals our results confirmed these findings, but not for small rural hospitals. In this case, our results show just the opposite in that inpatients admitted to urban hospitals in the region of Stara Zagora were more satisfied compared to inpatients in small rural hospitals.

The correlation analysis provided some additional information towards the relationship between overall satisfaction and patient satisfaction with different aspects of inpatient care. The results showed overall satisfaction of inpatients of the Stara Zagora region to be related to satisfaction with information about health-related behaviour after discharge, information about the treatment provided, achieved outcomes, and coordination of care. To some extent, overall satisfaction was also related to satisfaction with accommodation and nursing care.

In general, our results showed that providers of inpatient care in the region of Stara Zagora matched patients' expectations and fulfilled most of the patients' information needs. However, our results exhibited that positive or negative changes in satisfaction with particular aspects of inpatient care could influence overall satisfaction in the same direction. Comparable to findings of several international studies [2,31,32], our results suggest that in the Bulgarian population, overall inpatient satisfaction correlates predominately positive with information, education and coordination processes and somehow less with room comfort, attitude towards patient opinion and patients' participation in decision making.

Overall, our findings must be interpreted in light of the functionality of the Bulgarian health care system. First of all, Bulgarian patients have the choice of hospitals but are usually brought to the nearest hospital. In the standard case, patients are only familiar with services provided there and are not able to compare. Secondly, Bulgarians face a lot of rules restricting hospital admission and patients tend to be satisfied that they have been admitted to hospital at all.

Nevertheless, our results could be of use to stakeholders in health policy and hospital management in triggering quality improvement activities.

## Conclusion

The study demonstrates that questionnaires may be used to asses patient satisfaction with inpatient care in Bulgarian hospitals. Collecting the data by face-to-face contacts between researchers and patients generates high response rates. However, because of the high amount of resources in men-power, time and funds needed, this approach may not be feasible in routine practice.

The overall satisfaction of inpatients with acute coronary syndrome in Bulgaria is associated with the type of hospital, the number of family members living together and the severity of the disease at admission. According to these findings, the efforts by hospital managers to improve quality of care should target specific patient groups, for example women, patients living in small families and patients with less severe conditions at admission who showed to be less satisfied with their inpatient stay in general. In addition, the information obtained from the study could be used at decision-making level for implementing new strategies for structural changes in the Bulgarian inpatient health care system. To achieve a higher level of patient satisfaction, efforts to provide information and education, to improve coordination of care and to provide better accommodation should be undertaken. High cooperation of the patients indicates interest and willingness for changes from the patient's point of view. Bulgarian patients seem to be ready for the more patient-centered health care system, which Bulgarian health policy reforms have promised to strive for.

## Competing interests

The authors declare that they have no competing interests.

## Authors' contributions

MGI conceived and designed the study, and drafted the manuscript. KK performed data analysis and revised the manuscript critically for important intellectual content. MG contributed to conception and design of the study and revised the manuscript critically for important intellectual content. All authors read and approved the final manuscript.
